# Effects of Partial Replacing Fish Meal with Dietary Degossypolled Cottonseed Protein on Growth Performance, Intestinal Barrier, Immune Function and Antioxidant Response in Largemouth Bass (*Micropterus salmoides*)

**DOI:** 10.3390/ani16192997

**Published:** 2026-09-23

**Authors:** Jie Li, Songting Yang, Xunshang Zhang, Xiaotong Zhu, Hao Zhang, Mingxu Xie, Chenglong Wu

**Affiliations:** School of Life Science, Huzhou Normal University, 759 East 2nd Road, Huzhou 313000, China

**Keywords:** degossypolled cottonseed protein, fish meal replacement, largemouth bass, intestinal health, antioxidant capacity, inflammatory response

## Abstract

The increasing need for aquafeed protein sources calls for sustainable substitutes for fish meal (FM). Degossypolled cottonseed protein (DCP) represents a promising plant-derived protein, but its impact on the intestinal health of carnivorous fish is not well understood. This study assessed how partial replacing FM with DCP affects growth performance, intestinal barrier integrity, immune function, and antioxidant status in juvenile largemouth bass. Five isonitrogenous and isoenergetic diets were prepared, in which DCP replaced 0%, 20%, 40%, 60%, or 80% of the FM. Following an 8-week feeding period, the group receiving 20% DCP replacement showed no significant differences in growth or feed efficiency relative to the control, while 80% replacement caused marked growth reduction and increased visceral fat accumulation. High replacement levels (≥60%) downregulated antioxidant-related genes, damaged intestinal villi, compromised barrier integrity (mucins and tight junctions), and activated inflammatory responses via the NLRP3 inflammasome pathway, along with suppression of immune parameters. In conclusion, replacing 20% of FM with DCP was safe for largemouth bass, while excessive substitution (≥60%) induces intestinal oxidative stress, barrier dysfunction, inflammation, and immunosuppression. These findings support DCP as a viable partial substitute for FM in commercial diets for this species.

## Simple Summary

This study tested whether the degossypolled cottonseed protein (DCP) can safely replace fish meal in the diet of farmed largemouth bass (*Micropterus salmoides*). Fish meal is the traditional protein source for carnivorous fish, but it is expensive and its supply is limited. DCP is a more sustainable plant-based alternative. Juvenile bass were fed diets where DCP replaced 0%, 20%, 40%, 60%, or 80% of fish meal for eight weeks. The results showed that replacing up to 20% of fish meal with DCP did not harm fish growth or health. However, higher replacement levels (60% or more) caused problems, including slower growth, damage to the intestinal lining, reduced antioxidant defenses, gut inflammation, and weakened immune function. Based on these findings, a 20% replacement level is safe and effective for largemouth bass. This research helps fish farmers reduce reliance on fish meal by using a more sustainable plant-based protein, supporting more environmentally friendly aquaculture while maintaining fish health and growth.

## 1. Introduction

Fish meal (FM) is historically regarded as the premier protein ingredient in aquafeeds, attributed to its optimal profile of essential amino acids, abundant phospholipids and minerals, high palatability, and excellent digestibility [1]. Global FM production has remained stagnant for years, yet aquaculture demand continues to rise, driving prices upward [2]. This has pushed researchers to find cheaper, sustainable replacements. Plant proteins are one promising option. They contain high crude protein and good amino acid profiles. Because of this, many fish species now receive diets with plant proteins instead of FM [3]. Soybean meal, cottonseed meal, corn gluten meal, canola meal, guar meal, sunflower meal, almond meal, lupins, rice, and wheat are all common choices [4]. Despite their value as sources of macronutrients and micronutrients, plant proteins contain abundant antinutritional factors (ANFs), which restrict their application in aquafeeds [5]. When included in fish diets, these ANFs can induce multiple adverse effects—such as impaired growth, reduced nutrient utilization efficiency, intestinal dysfunction, altered immune responses, and shifts in gut microbiota [6,7].

Cottonseed meal, a plant protein with a balanced amino acid profile, is a promising alternative protein source to FM. Many studies have shown that partial replacing FM with cottonseed meal has no negative impacts on the growth, health and antioxidant parameters of fish such as largemouth bass (*Micropterus salmoides*) [8], Nile tilapia (*Oreochromis niloticus*) [9], rohu (*Labeo rohita*) [10], golden pompano (*Trachinotus ovatus*) [11], pearl gentian groupers (*Epinephelus fuscoguttatus*♀ × *E. lanceolatus*♂) [12] and black sea bream (*Acanthopagrus schlegelii*) [13]. However, the utilization of cottonseed meal in aquaculture is severely limited by the presence of free gossypol, a yellow polyphenolic aldehyde that is the most harmful to monogastric animals, including fish species [14]. Detoxification technologies that remove or inactivate free gossypol can transform cottonseed meal into a high-quality protein—specifically, degossypolled cottonseed protein (DCP)—thereby producing a product that is distinct from conventional cottonseed protein concentrate (CPC) in terms of its markedly reduced free gossypol content and improved protein quality, offering a sustainable alternative to FM. Numerous studies support this potential. For instance, Liu et al. (2020) demonstrated that supplementing low-gossypol cottonseed meal with amino acids enabled it to replace 16% of FM for silver sillago (*Sillago sihama* Forsskál) (1775) without affecting growth or feed utilization compared with the FM-based control [14]. Similarly, this study by Zhang et al. (2022) showed that low gossypol cottonseed meal could replace up to 35% FM in the diet of juvenile turbot (*Scophthalmus maximus* L.), with no adverse effects on growth performance, feed utilization and haematological responses [15]. Notably, replacing 10–50% of FM with concentrated dephenolized cottonseed protein can enhance growth, hematology, antioxidant status, and body composition of rainbow trout (*Oncorhynchus mykiss*) [16]. While previous studies on cottonseed products have primarily focused on growth performance, feed utilization, and systemic hematological and antioxidant parameters, the specific impacts of DCP—particularly its low-gossypol formulation—on intestinal health in carnivorous fish remain poorly understood.

The largemouth bass (*Micropterus salmoides*), native to North America, has become a major farmed fish in Asia, especially in China [17,18]. Valued for its distinctive flavor and straightforward cultivation requirements, the industry has experienced steady growth [19]. China now produces about 93,000 tons per year, up 5.0% from last year (China Fishery statistical yearbook, 2025). As a carnivorous fish, the largemouth bass requires a diet high in protein. Although FM has long been the primary protein source, its high cost and limited availability make it necessary to search for alternative protein sources to reduce production costs and enhance the sustainability of aquaculture [20]. Carnivorous teleosts such as largemouth bass possess a relatively short digestive tract and rapid gastrointestinal transit, rendering their intestinal epithelium, local mucosal immune networks, and endogenous antioxidant defense systems particularly susceptible to dietary perturbations and antigenic challenges. Consequently, any alterations in dietary protein composition may exert profound impacts on intestinal barrier integrity, immune homeostasis, and redox balance in this species [21,22]. This study was therefore designed to test the hypothesis that partial replacement of FM with DCP, at appropriate inclusion levels, would improve or at least maintain intestinal barrier function, immune homeostasis, and antioxidant capacity in juvenile largemouth bass, whereas excessive DCP inclusion would compromise these parameters through the induction of oxidative stress, inflammatory responses, and disruption of tight junction integrity. Unlike previous research that has largely concentrated on conventional CPC or whole cottonseed meal, the present work specifically focuses on the degossypolled product (DCP) to evaluate its effects on intestinal health. To our knowledge, it represents the first investigation into the potential benefits of graded DCP inclusion on the gut health of largemouth bass. Ultimately, these findings are expected to support the application of DCP as a viable aquafeed supplement for this commercially important species.

## 2. Materials and Methods

### 2.1. Experimental Diets and Formulation

Degossypolled cottonseed protein (DCP, 64% crude protein) came from Xinjiang Chengrun Jinlan Biotechnology Co., Ltd. (Kuqa, Xinjiang, China). The residual free gossypol content in the DCP product was approximately 150 mg/kg, as determined by the manufacturer’s analysis. An FM-based basal diet served as the control. In the treatment diets, FM was replaced by DCP at 0%, 20%, 40%, 60%, or 80% (designated DCP0–DCP80), giving DCP inclusion rates of 0%, 13.2%, 26.4%, 39.6%, and 52.8%, respectively. All five diets were isonitrogenous (47.5% crude protein) and isoenergetic (10% crude lipid). The minor variations in analyzed crude protein (48.13–48.79%) and crude lipid (11.18–11.37%) shown in Table 1 represent acceptable analytical deviations, while the base formulation matrix remained strictly balanced. Table 1 provides the ingredient composition, analyzed lysine and methionine contents, and other nutrient levels of each diet. All ingredients were ground (60 mesh), weighed, and blended thoroughly. The mash was then extruded into 2.5 mm pellets using a twin-screw extruder. Pellets were oven-dried at 37 °C with air circulation and stored at 4 °C until use.

### 2.2. Fish Rearing and Feeding Trial

Healthy juvenile largemouth bass purchased from Zhengda Aquatic Products (Huzhou) Co., Ltd. (Huzhou, China) were first acclimated in glass aquaria. A total of 450 fish (initial weight: 3.45 ± 0.05 g) were randomly allocated to 5 treatment groups, each with 3 replicate tanks (30 fish/tank). Each group was fed one of the experimental diets to satiation twice per day (07:00 and 17:00). The daily feed intake for each tank was recorded as the total amount of feed offered. Throughout the 8-week trial, all offered feed was consistently consumed within 15–20 min without observable leftovers; hence, no uneaten feed recovery was required. During the 8-week feeding period, water temperature was kept near 28 °C, pH ranged from 6.8 to 7.2, and dissolved oxygen remained above 5 mg/L. Fish feeding behavior was observed daily.

### 2.3. Sampling Collection and Biometric Measurements

Following the 8-week feeding period, fish were fasted for 24 h. All largemouth bass per tank were counted and bulk-weighed. Five individuals per tank were randomly taken, weighed singly, and frozen at −20 °C for whole-body composition analysis. A further ten fish per tank were randomly chosen and weighed individually. Blood was collected from the caudal vein into 1.5 mL centrifuge tubes. After being held at 4 °C for 8 h, the blood was centrifuged at 2500 rpm for 10 min, and the plasma supernatant was separated, aliquoted, and stored at −80 °C. Following blood sampling, each fish was dissected to isolate the viscera (including the gastrointestinal tract, spleen, and kidney), liver, intestinal fat, and abdominal fat. The weight of each tissue was recorded to compute the viscerosomatic index (VSI) and hepatosomatic index (HSI). The liver was weighed for hepatosomatic index (HSI) calculation and was not subjected to further biochemical analyses; the intestinal fat and abdominal fat were used only for index calculation and were not subjected to further biochemical analyses. Intestinal tissues were collected and processed according to the following scheme: proximal intestine was used for digestive enzyme activity assays; mid-intestine was fixed in 4% paraformaldehyde for histological examination; distal intestine was snap-frozen in liquid nitrogen and stored at −80 °C for RNA extraction to analyze antioxidant, barrier, inflammatory, and immune-related gene expression. All subsequent measurements were performed on these pooled samples, yielding one biological datum per tank. Thus, the tank (*n* = 3 replicate tanks per treatment) was treated as the true independent experimental unit, thereby avoiding pseudo-replication.

### 2.4. Growth Performance Indices

Several growth and health-related parameters were determined using the formulas outlined below:

Weight gain rate (WG, %): WG = [(Final body weight − Initial body weight)/Initial body weight] × 100

Specific growth rate (SGR, %/d): SGR = [(ln Final body weight − ln Initial body weight)/Experimental days] × 100

Feed conversion ratio (FCR): FCR = Feed intake/(Final body weight − Initial body weight)

Hepatosomatic index (HSI, %): HSI = (Liver weight/Body weight) × 100

Intraperitoneal fat ratio (IPR, %) = (Intraperitoneal fat weight/Body weight) × 100

Condition factor (CF, g/cm^3^): CF = (Body weight/Body length^3^) × 100

### 2.5. Proximate Composition Analysis

The proximate composition of the experimental diets and fish samples (whole body) was analyzed according to standard methods described in “Feed Analysis and Testing”. The analyzed components included dry matter, moisture, crude protein, crude lipid, and ash. To determine moisture, specimens were oven-dried at 105 °C for 16 h, and the moisture level was derived from the weight difference before and after drying. Crude protein was assayed using the Dumas combustion technique. Crude lipid was estimated by Soxhlet extraction. Ash was quantified based on the residue weight following muffle furnace incineration at 550 °C for 3 h, in line with AOAC (2000) procedures.

### 2.6. Measurement of Serum Biochemical Parameters 

Levels of high-density lipoprotein cholesterol (HDL-C), low-density lipoprotein cholesterol (LDL-C), alanine aminotransferase (ALT), aspartate aminotransferase (AST), alkaline phosphatase (ALP), glucose (GLU), total bile acids (TBA), albumin (ALB), blood urea nitrogen (BUN), and lactate dehydrogenase (LDH) were determined using commercial kits supplied by Ningbo Prebon Biotechnology Co. (Ningbo, China). Each assay was performed with a minimum of three replicates.

### 2.7. Measurement of Physiological and Biochemical Indexes

The activities of α-amylase (α-AMS), trypsin (TRY), and chymotrypsin (CYT) were assessed using commercial assay kits obtained from Jiancheng Biotech (Nanjing, China), strictly following the manufacturer’s protocols. Additionally, serum levels of complement C3, complement C4, immunoglobulin M (IgM), alkaline phosphatase (AKP), alkaline phosphatase (ALP), and lysozyme (LZM) were quantified using kits supplied by the same company. All biochemical measurements were performed in at least triplicate to ensure reliability and reproducibility of the data.

### 2.8. Quantitative Real-Time PCR Analysis

Intestinal tissue samples from largemouth bass were homogenized in TRIzol reagent (Invitrogen, Carlsbad, CA, USA) to isolate total RNA. RNA purity and integrity were assessed by spectrophotometry and agarose gel electrophoresis. Genomic DNA contamination was removed via DNase I treatment (Takara, Dalian, China). Subsequently, 10 μg of purified RNA was reverse-transcribed into cDNA using Super Script™ II Reverse Transcriptase (Takara, Dalian, China) in a 75 μL reaction mixture. Primer pairs targeting immune-related genes were designed based on largemouth bass genomic sequences (Table 2) and synthesized by Biosune Co. (Shanghai, China). The qPCR runs were conducted under standard thermal cycling conditions. Relative transcript levels were normalized to the internal control *β-actin* and determined by the 2^−ΔΔCt^ method.

### 2.9. Statistical Analysis

All experimental results were processed using one-way ANOVA in SPSS (version 25.0). To identify statistically meaningful differences between groups, Duncan’s multiple range test was applied, with significance determined by a *p*-value threshold. The final data are expressed as mean ± standard deviation (mean ± SD).

## 3. Results

### 3.1. Growth Performance, Feed Utilization, and Body Composition

Table 3 indicates that replacing FM with 20% DCP caused no significant differences in final body weight (FBW) or WGR versus the control (*p* > 0.05). The lowest FBW and WGR were recorded at 80% substitution. FBW and WGR in the DCP0, DCP20, and DCP40 groups were comparable to each other (*p* > 0.05), and all three groups were significantly higher than those in the DCP60 group (*p* < 0.05). The DCP60 group, in turn, was significantly higher than the DCP80 group (*p* < 0.05). For SGR, no significant change was seen at 20% substitution (*p* > 0.05), but SGR fell significantly from 40% substitution onward (*p* < 0.05). FCR did not differ significantly among the control, 20%, 40%, and 60% groups (*p* > 0.05), yet the 80% group had a significantly higher FCR than the control (*p* < 0.05). The 80% group also had the highest VSI, significantly above all other groups (*p* < 0.05), with no other differences in VSI. Intraperitoneal fat ratio (IPR) was significantly increased relative to the control (*p* < 0.05). HSI and CF were not significantly affected by DCP replacement level (*p* > 0.05). Among all replacement levels, the DCP20 group exhibited the highest WGR and SGR values, which were statistically comparable to those of the control group (*p* > 0.05), whereas higher substitution levels (≥60%) resulted in significant growth depression (*p* < 0.05). The optimal level of DCP to replace FM, based on WGR as the evaluation indicator, was found to be 8.11% through quadratic regression curve analysis (Figure 1).

Dietary DCP substitution also significantly affected whole-body composition (Table 3). Moisture content was significantly higher in the DCP80 group than in the control group (*p* < 0.05). Crude lipid content showed an increasing trend with rising DCP levels, with the DCP80 group exhibiting a significantly higher value than the control (*p* < 0.05). Ash content remained comparable to the control at the 20% substitution level (*p* > 0.05), but decreased significantly in the DCP40, DCP60, and DCP80 groups in a dose-dependent manner (*p* < 0.05). No significant differences were observed in whole-body crude protein content among any of the groups (*p* > 0.05).

### 3.2. Intestinal Digestive Enzyme Activities and Related Gene Expression

According to Table 4, dietary FM substitution with DCP significantly altered intestinal digestive enzyme activities. α-Amylase (AMS) activity was significantly reduced in the 40% and 80% groups versus the control (*p* < 0.05), but significantly increased in the 20% group (*p* < 0.05). The 60% group did not differ from either the control or the 20% group, and the lowest AMS value was found at 80% substitution. For trypsin (TRY), no significant differences were seen in the 60% or 80% groups relative to the control, while the 20% and 40% groups showed significantly higher activity (*p* < 0.05), with the 20% group being the highest. For chymotrypsin (CYT), the 40%, 60%, and 80% groups were comparable to the control. The 20% group had significantly higher CYT than the control and the 80% group (*p* < 0.05), but was not significantly different from the 40% or 60% groups.

### 3.3. Serum Biochemical Parameters

According to Table 5, HDL-C did not vary notably among DCP groups except that the 80% group was significantly higher than the 40% group (*p* < 0.05). LDL-C was significantly lower in the 60% DCP group versus the 0% DCP group (*p* < 0.05). AST activities showed no significant group differences (*p* > 0.05), while ALT activities were significantly higher in the 60% and 80% groups than in lower substitution groups (*p* < 0.05). ALP activity was significantly elevated in the 0% DCP group compared to all DCP-containing groups (*p* < 0.05). GLU content declined with increasing DCP, with the 0% group significantly higher than all replacement groups, and the 40% and 80% groups recording the lowest levels (*p* < 0.05). TBA remained unchanged up to 60% substitution but rose significantly at 80% DCP (*p* < 0.05). ALB levels were similar from 0% to 60% DCP but dropped significantly at 80% DCP (*p* < 0.05). BUN trended upward, with the 60% DCP group significantly higher than the 0% and 40% groups (*p* < 0.05). LDH activity generally increased, and the 80% DCP group was significantly higher than the 0% and 20% groups (*p* < 0.05).

### 3.4. Intestinal Antioxidant-Related Gene Expression

Figure 2 illustrates that superoxide dismutase 1 (*SOD1*) transcript levels decreased significantly with increasing DCP inclusion (*p* < 0.05). *SOD1* expression was significantly higher in the DCP0 and DCP20 groups than in the DCP40 and DCP80 groups (*p* < 0.05). No notable differences in *SOD1* were found among the DCP40, DCP60, and DCP80 groups (*p* > 0.05), whereas the DCP0 group had significantly higher expression than the DCP20 group (*p* < 0.05). For superoxide dismutase 2 (*SOD2*), the DCP0, DCP20, DCP60, and DCP80 groups did not differ significantly (*p* > 0.05), but the DCP40 group showed a marked decrease relative to the DCP0 group (*p* < 0.05).

Glutathione peroxidase 4α (*Gpx4α*) expression was significantly reduced in all DCP-substituted groups (DCP20–DCP80) compared with the DCP0 group (*p* < 0.05). The highest *Gpx4α* value occurred in the DCP0 group and the lowest in the DCP80 group (*p* < 0.05), with no significant difference between the DCP40 and DCP60 groups (*p* > 0.05). Moreover, DCP replacement caused a significant downregulation of intestinal glutathione reductase (*GR*) expression versus the control (*p* < 0.05); the DCP0 group had the highest *GR* level and the DCP80 group the lowest (*p* < 0.05). Regarding glutathione synthase (*Gss*), no significant differences were observed among the DCP0, DCP20, DCP40, and DCP80 groups (*p* > 0.05), except that the DCP60 group showed a significant downregulation compared to the DCP0 group (*p* < 0.05).

Glutamate-cysteine ligase catalytic subunit (*Gclc*) expression was significantly elevated in the DCP0 and DCP20 groups relative to the DCP40, DCP60, and DCP80 groups (*p* < 0.05), with no notable difference between DCP0 and DCP20 (*p* > 0.05). The lowest *Gclc* value was in DCP80, although no significant differences were seen among DCP40, DCP60, and DCP80 (*p* > 0.05). Glutamate-cysteine ligase modifier subunit (*Gclm*) showed a significant dose-dependent decline with increasing DCP substitution (*p* < 0.05). DCP0 had the highest *Gclm* level, significantly above all others (*p* < 0.05). DCP20 also had significantly higher *Gclm* than DCP60 and DCP80 (*p* < 0.05), and DCP80 had the lowest level (*p* < 0.05).

Conversely, glutathione S-transferase A (*GST-A*) expression increased markedly with rising DCP inclusion, reaching its maximum in DCP80 (*p* < 0.05), with no significant difference between DCP40 and DCP60 (*p* > 0.05). Thioredoxin (*Trx*) expression was highest in DCP0 and lowest in DCP80 (*p* < 0.05). Thioredoxin reductase (*TrxR*) expression was significantly higher in DCP0 than in all other groups (*p* < 0.05), with DCP80 again showing the lowest level (*p* < 0.05). No significant *TrxR* difference was found between DCP60 and DCP80 (*p* > 0.05), but DCP20 had significantly higher *TrxR* expression than DCP40, DCP60, and DCP80 (*p* < 0.05).

Overall, DCP replacement at ≥60% significantly suppressed the expression of most antioxidant genes (*SOD1*, *Gpx4α*, *GR*, *Gclc*, *Gclm*, *Trx*, and *TrxR*) in the intestine, while *GST-A* expression was significantly induced. These findings indicate that excessive DCP substitution may impair intestinal antioxidant capacity by reducing key enzyme transcripts, and the upregulation of *GST-A* may serve as a compensatory mechanism against DCP-induced oxidative stress in largemouth bass.

### 3.5. Intestinal Histomorphology

Dietary DCP substitution significantly influenced intestinal morphology (Table 6; Figure 3). Villus height and villus width remained relatively stable across all treatments. Muscular thickness decreased progressively with increasing DCP levels, with the DCP80 group showing the lowest value (*p* < 0.05). Crypt depth initially decreased at DCP20 but increased at DCP40 and DCP60, then decreased again at DCP80. Goblet-cell count per villus decreased progressively with increasing DCP substitution. Specifically, the DCP0 and DCP20 groups exhibited significantly higher goblet-cell counts compared with the DCP40, DCP60, and DCP80 groups (*p* < 0.05), with no significant difference observed among the three higher substitution groups (*p* > 0.05). These results suggest that excessive DCP substitution (≥60%) negatively affects intestinal structural integrity in largemouth bass.

### 3.6. Intestinal Barrier-Related Gene Expression

As shown in Figure 4, *Mucin-2* expression did not differ significantly among the DCP0, DCP20, DCP40, and DCP60 groups (*p* > 0.05), but was significantly lower in the DCP80 group than in the other groups (*p* < 0.05). *Mucin-5AC* exhibited a decreasing trend, with the 40%, 60%, and 80% groups significantly below the control (*p* < 0.05). *Mucin-13* declined progressively with increasing DCP levels (*p* < 0.05). No significant difference existed between DCP0 and DCP20 (*p* > 0.05), but both were significantly higher than DCP40, DCP60, and DCP80 (*p* < 0.05). For *Mucin-17*, the DCP0, DCP20, and DCP40 groups had relatively higher expression than the DCP80 group (*p* < 0.05).

For tight junction genes, Claudin 15 (*Cldn15*) was significantly downregulated in all replacement groups versus the control (*p* < 0.05), with no differences among replacement levels (*p* > 0.05). Claudin 34 (*Cldn-34*) expression was significantly higher in DCP0 and DCP20 than in DCP60 and DCP80 (*p* < 0.05), peaking in DCP0 and bottoming in DCP80 (*p* < 0.05). *Occludin 1* expression was significantly higher in DCP0, DCP20, and DCP40 than in DCP60 and DCP80 (*p* < 0.05), with no differences among the first three groups (*p* > 0.05). The lowest *Occludin 1* level was in DCP80, with no significant difference between DCP60 and DCP80 (*p* > 0.05).

In summary, DCP replacement at ≥60% significantly suppressed most mucus barrier genes (*Mucin-5AC*, *Mucin-13* and *Mucin-17*) and tight junction genes (*Cldn15*, *Cldn-34*, and *Occludin 1*). These results indicate that excessive DCP substitution may compromise intestinal barrier function via reduced mucus production and altered tight junction protein expression.

### 3.7. Intestinal Inflammation-Related Gene Expression

As shown in Figure 5, interleukin-8 (*IL-8*) transcripts rose steadily with increasing DCP substitution, peaking in DCP80 (*p* < 0.05). No significant difference existed between DCP0 and DCP20 (*p* > 0.05), but DCP60 and DCP80 had significantly higher *IL-8* than both DCP0 and DCP20 (*p* < 0.05). Interleukin-1β (*IL-1β*) and interleukin-12β (*IL-12β*) were elevated in DCP40, DCP60, and DCP80 versus DCP0 and DCP20 (*p* < 0.05), with no differences among DCP40–DCP80 (*p* > 0.05). Caspase-1 (*Cas-1*) increased gradually with higher DCP replacement, peaking in DCP80 (*p* < 0.05). DCP60 and DCP80 had markedly higher Cas-1 than DCP0 and DCP20 (*p* < 0.05), with no difference between DCP0 and DCP20 (*p* > 0.05). Caspase-6 (*Case-6*) showed a dose-dependent upward trend (*p* < 0.05), with DCP80 highest among all groups (*p* < 0.05) and DCP60 also significantly above DCP0 and DCP20 (*p* < 0.05).

Gasdermin E (*GSDM-E*) did not differ significantly among DCP0–DCP60 (*p* > 0.05), though DCP80 was slightly higher than DCP0 (*p* < 0.05). NOD-like receptor family pyrin domain containing 3 (*NLRP3*) was dramatically elevated in DCP80 versus all others (*p* < 0.05), and DCP60 was also significantly above DCP0 (*p* < 0.05), with no changes among DCP0, DCP20, and DCP40 (*p* > 0.05). Nucleotide-binding oligomerization domain-containing protein 1 (*NOD1*) increased with rising DCP, peaking in DCP80 (*p* < 0.05). DCP60 and DCP80 had notably higher *NOD1* than DCP0 and DCP20 (*p* < 0.05), with no differences among DCP0–DCP40 (*p* > 0.05). Apoptosis-associated speck-like protein containing a CARD (*ASC*) was significantly higher in DCP80 than in all others (*p* < 0.05), and DCP60 also showed markedly elevated expression versus DCP0 and DCP20 (*p* < 0.05). In summary, DCP replacement at ≥60% significantly upregulated most inflammatory genes (*IL-8*, *Cas-1*, *Case-6*, *NLRP3*, *NOD1*, and *ASC*), indicating that excessive DCP substitution may trigger inflammatory responses via activation of the NLRP3 inflammasome pathway.

### 3.8. Intestinal Immune-Related Gene Expression and Humoral Immune Parameters

Table 7 shows that the extent of FM replacement by DCP significantly modulated the intestinal humoral immune parameters of largemouth bass. At the 20% substitution level, most immune parameters—including complement C3, C4, immunoglobulin M (IgM), acid phosphatase (ACP), and lysozyme (LZM)—remained comparable to the control group (*p* > 0.05), with the exception of alkaline phosphatase (AKP) activity, which was significantly reduced even at this level (*p* < 0.05). Higher substitution levels (40–80%) led to more pronounced immunomodulatory effects. Specifically, C3, IgM, and ACP levels were significantly lower in the 60% and 80% groups (*p* < 0.05). C4 content was significantly reduced in the 40%, 60%, and 80% groups (*p* < 0.05). AKP activity declined in a dose-dependent manner as DCP inclusion increased. LZM activity was significantly decreased at 40% and 80% substitution compared with the control (*p* < 0.05). Taken together, a 20% replacement level had minimal impact on most intestinal humoral immune factors, whereas substitution exceeding this threshold triggered notable immunosuppressive effects.

Figure 6 shows that FM replacement by DCP significantly affected intestinal immune gene expression. Hepcidin (*HEPC*) decreased significantly with rising DCP levels (*p* < 0.05). DCP0 had the highest *HEPC*, significantly above all others (*p* < 0.05); DCP20 was also significantly higher than DCP40–DCP80 (*p* < 0.05); DCP80 had the lowest value (*p* < 0.05). Lysozyme (*LZM*) was significantly higher in DCP20 and DCP40 than in DCP0, DCP60, and DCP80 (*p* < 0.05), peaking in DCP20 and bottoming in DCP80 (*p* < 0.05), with no difference between DCP20 and DCP40 (*p* > 0.05). Immunoglobulin light chain (*Ig-L*) was significantly higher in DCP0 and DCP20 than in DCP60 and DCP80 (*p* < 0.05), highest in DCP20 and lowest in DCP80 (*p* < 0.05). DCP0 and DCP40 did not differ (*p* > 0.05), but DCP80 was significantly lower than DCP40 (*p* < 0.05). Immunoglobulin M (*Ig-M*) dropped considerably in DCP60 and DCP80 versus DCP0 and DCP20 (*p* < 0.05), with DCP0 highest and DCP80 lowest (*p* < 0.05), and no difference between DCP0 and DCP20 (*p* > 0.05). Tissue factor pathway inhibitor 2 (*Tfpi2*) was significantly higher in DCP0, DCP20, and DCP40 than in DCP60 (*p* < 0.05), with no differences among the first three (*p* > 0.05). DCP60 had the lowest *Tfpi2* (*p* < 0.05), and DCP80 was also significantly lower than DCP0 and DCP20 (*p* < 0.05).

Selenoprotein F (*SEPF*) was significantly higher in DCP0 than in DCP40–DCP80 (*p* < 0.05); DCP20 was intermediate, not different from DCP0 (*p* > 0.05) but significantly above DCP60 and DCP80 (*p* < 0.05). Selenoprotein K (*SEPK*) was much higher in DCP0 and DCP20 than in DCP40–DCP80 (*p* < 0.05), peaking in DCP0 and bottoming in DCP80 (*p* < 0.05), with no difference between DCP0 and DCP20 (*p* > 0.05). Selenoprotein S (*SEPS*) was significantly higher in DCP0 than in DCP40–DCP80 (*p* < 0.05); DCP40–DCP80 did not differ among themselves (*p* > 0.05), while DCP20 was significantly higher than DCP60 and DCP80 (*p* < 0.05). Overall, DCP replacement at ≥60% significantly downregulated most immune genes (*HEPC*, *Ig-L*, *Ig-M*, *Tfpi2*, *SEPF*, *SEPK*, *SEPS*), while *LZM* was upregulated at 20–40% DCP. These results suggest that excessive DCP substitution may impair intestinal immune defense by reducing antimicrobial peptides, immunoglobulins, and cytoskeletal regulators, whereas moderate DCP levels may enhance innate immunity via *LZM* upregulation.

## 4. Discussion

### 4.1. Growth Performance and Body Composition

Fish growth performance is a key indicator in evaluating plant protein as an alternative to FM. In this study, the replacement level of FM with DCP significantly influenced the growth of largemouth bass. The 20% DCP Supplementation showed no adverse effects on FBW, WGR, SGR or FCR, suggesting that DCP can be effectively utilized as a partial protein alternative at this inclusion level. These results are consistent with reports in other fish species, where moderate levels of protein sources are generally well-tolerated without compromising growth [23,24]. In contrast, over-substitution of cottonseed meal products has been found to inhibit the growth of aquatic animals, with the ideal inclusion level differing among species [14,25]. In this work, feed conversion efficiency stayed stable up to a 60% substitution level of DCP, as indicated by the unchanged FCR. Nevertheless, a significant drop in growth accompanied by a sharp increase in FCR at the 80% replacement level suggests an exceeded tolerance limit. Although the raw DCP ingredient contained a low level of free gossypol (~150 mg/kg), its exact concentration in the finished diets was not analytically quantified. Therefore, the adverse effects observed at the highest substitution level cannot be definitively attributed to gossypol toxicity. Instead, a more plausible alternative explanation is the limitation of essential amino acids. Plant proteins, particularly cottonseed products, are classically deficient in lysine and methionine compared to high-quality fish meal. As indicated in Table 1, the dietary lysine and methionine levels decreased with increasing DCP inclusion. Largemouth bass have specific requirements for these amino acids to support optimal protein synthesis and muscle accretion. Therefore, the significant growth depression and decreased feed efficiency observed at the 60% and 80% replacement levels are likely driven to a large extent by lysine limitation, which restricts overall protein utilization, acting as a major limiting factor independent of gossypol or other anti-nutritional factors [14,26,27]. The exact underlying mechanism remains to be explored, combined with the amino acid profile and fatty acid content in these different treated groups.

Digestive enzymes are key to how effectively animals convert feed into absorbable nutrients, which in turn shapes their energy intake and growth performance [28]. Our findings showed that a 20% DCP level increased the activities of AMS, TRY, and CYT, whereas 80% DCP lowered AMS activity. In line with these enzymatic shifts, low DCP inclusion (20% and 40%) upregulated genes related to digestive enzymes, unlike the 80% DCP level, which had no significant effect. Similar results were also shown in black carp (*Mylopharyngodon piceus*) [29] and the Pacific white shrimp (*Litopenaeus vannamei*) [30]. Combined with the growth data, DCP supports normal growth in largemouth bass when used as appropriate FM replacement levels, likely through modulating digestive enzyme activity. Considering the growth performance data (WGR and SGR) together with the intestinal health indicators, a 20% replacement level of FM with DCP appears to be the optimal inclusion rate for juvenile largemouth bass, as it maintains growth without compromising intestinal barrier integrity, antioxidant capacity, or immune function.

In addition to growth, the physiological responses offer further insight into health status. In the present study, fish receiving 80% DCP showed a notable rise in both VSI and IPR, pointing to a clear accumulation of energy reserves, especially as visceral fat. VSI and IPR serve as important quantitative indicators in fish biology [31,32]. VSI reflects the relative weight of visceral organs, while IPR specifically quantifies fat deposition within the body cavity [33,34]. Importantly, neither the HSI or CF changed significantly, suggesting that this fat accumulation was largely confined to the visceral cavity, without causing liver enlargement or altering overall body shape and muscle mass. The whole-body composition data further supported the observed changes in lipid metabolism. The increased crude lipid content and decreased ash content in the high-DCP groups (≥60%) are consistent with the elevated IPR observed in these fish, suggesting that excessive DCP substitution may promote lipid deposition while reducing mineral retention. The unchanged crude protein content across all groups indicates that protein deposition was relatively stable despite the growth reduction at high DCP levels, implying that the growth depression observed at 80% substitution may be primarily due to reduced feed intake or nutrient utilization efficiency rather than impaired protein synthesis *per se*.

### 4.2. Serum Biochemical Responses

Serum biochemical indices serve as crucial biomarkers for assessing the metabolic capacity and overall health of farmed fish. Alterations in serum lipid parameters, including HDL-C and LDL-C, mirror the metabolic and physiological condition of fish and are widely employed to evaluate lipid metabolism [35]. Furthermore, the rise in HDL-C at 80% DCP along with lower LDL-C at 60% indicates a possible improvement in cholesterol transport at moderate to high replacement levels, although the physiological role of lipoprotein metabolism in fish needs further study.

ALT is considered a biomarker of hepatic injury and chronic hepatitis; an increase in its plasma activity preliminarily suggests impaired liver function [36]. ALP serves as a biomarker for obstructive jaundice and cholestasis, and is also a key contributor to hepatic fibrosis; a significant elevation in ALP activity typically indicates the presence of cholestasis [37]. In this experiment, plasma ALT activity rose in the 60% and 80% DCP groups, hinting at impaired liver function in largemouth bass. Meanwhile, the marked decrease in ALP activity suggests that cholestatic issues were not present in these fish. A notable increase in total bile acids (TBA) occurred only at the 80% substitution level, which aligns with the ALT results and points to possible alterations in bile acid metabolism or hepatic secretion at this high inclusion rate. Taken together, the elevated ALT and TBA raise concerns about liver health when DCP replaces 80% of FM.

Blood glucose (GLU) exhibited a non-monotonic pattern across increasing DCP substitution levels, with values of 6.99, 5.04, 3.13, 4.59, and 3.19 mmol/L for the DCP0, DCP20, DCP40, DCP60, and DCP80 groups, respectively. Specifically, GLU decreased progressively from DCP0 to DCP40, then increased at DCP60 before dropping again at DCP80. This fluctuating pattern suggests that DCP inclusion may influence carbohydrate metabolism or gluconeogenesis in a non-linear manner, possibly involving compensatory endocrine responses or changes in carbohydrate digestibility at intermediate inclusion levels [38]. A similar decline has been seen in fish fed soybean protein concentrate, although the non-monotonic rebound observed in the present study warrants further investigation [39]. Albumin (ALB), which serves as a key indicator of protein synthesis and nutritional status, stayed unchanged up to the 60% substitution level but fell sharply at 80%, suggesting that protein utilization or liver synthesis might only become compromised at the highest inclusion rate [40]. Blood urea nitrogen (BUN), which reflects protein catabolism and kidney performance [41], increased with increasing DCP levels and reached its highest point at 60% replacement, possibly reflecting higher nitrogen turnover or a temporary adaptation in ammonia detoxification following FM substitution. Elevated lactate dehydrogenase (LDH) activity, especially at 80% DCP, also suggests a shift in muscle energy metabolism, likely toward increased anaerobic glycolysis [42]. This could represent either a metabolic adjustment to the altered diet or a sign of physiological stress.

### 4.3. Intestinal Antioxidant Capacity

The intestine mediates nutrient digestion and absorption, while its antioxidant capacity is equally critical for maintaining epithelial integrity and supporting fish health [43]. In our work, replacing FM with DCP at levels of 60% or higher led to a significant decrease in most antioxidant-related genes in the gut of largemouth bass. One exception was GST-A, whose expression was markedly upregulated at higher DCP inclusion. These transcriptional changes may suggest that too much DCP could potentially impair the intestinal antioxidant defense system by suppressing key antioxidant enzymes, while simultaneously upregulating GST-A possibly as a compensatory reaction to counteract oxidative stress induced by high dietary DCP. Redox homeostasis is recognized as critical for normal gut function and immune enhancement in animals [44]. As the first defense against reactive oxygen species (ROS), superoxide dismutase (SOD) converts superoxide anion radicals to O_2_ and H_2_O_2_ [45]. In our study, SOD1 expression decreased significantly as DCP substitution increased. By contrast, SOD2 expression did not follow a monotonic dose-dependent pattern; instead, it remained relatively stable across most groups, with a notable decline observed only at the DCP40 substitution level, while recovering at DCP60 and DCP80. This non-monotonic response indicates that the mitochondrial isoform SOD2 may be differentially regulated compared with its cytoplasmic counterpart, possibly reflecting distinct sensitivities to oxidative stress or antinutritional factors at specific inclusion levels. These observations partially mirror earlier findings in swimming crab (*Portunus trituberculatus*) fed low-gossypol cottonseed protein concentrate [45] and in juvenile largemouth bass given a high-cottonseed protein diet [46], where higher levels of plant-based protein sources lowered SOD activity. The different behaviors of SOD1 and SOD2 may stem from their distinct locations within the cell—SOD1resides mainly in the cytoplasm, while SOD2 is found in mitochondria—as well as their unequal susceptibility to dietary antinutritional factors [47]. The non-monotonic nature of SOD2 expression, however, suggests that the relationship between DCP inclusion and mitochondrial antioxidant defense may be more complex than a simple linear dose-response, and warrants further mechanistic exploration.

Glutathione peroxidase 4α (Gpx4α) is a key antioxidant enzyme that uses glutathione (GSH) to reduce hydrogen peroxide and lipid hydroperoxides to alcohols, limiting oxidative damage [48,49]. Here, all DCP groups had significantly lower Gpx4α expression than the control, with the lowest level in DCP80. This matches previous reports in *Penaeus vannamei* fed high-plant-protein diets [50] and in Nile tilapia (*Oreochromis niloticus*) fed high plant protein levels [51], where higher dietary plant protein reduced GPx activities. The marked downregulation of *Gpx4α* at higher DCP levels may indicate that the glutathione-dependent antioxidant system could be severely impaired, which might lead to accumulation of lipid peroxides and potential oxidative damage to intestinal epithelial cells. Glutathione reductase (*GR*) and glutamate-cysteine ligase (*GCL*, including *Gclc* and *Gclm*) are key enzymes involved in GSH metabolism [48,49]. Higher DCP substitution progressively decreased *GR*, *Gclc*, and *Gclm* expression in our study, with the lowest levels occurring in the DCP80 group. The simultaneous drop in *GR*, *Gclc*, and *Gclm* may suggest that excessive DCP substitution not only reduces GSH regeneration but also inhibits GSH synthesis. This could potentially result in GSH depletion and a compromised antioxidant barrier in the gut of largemouth bass.

Interestingly, glutathione S-transferase A (*GST-A*) expression showed a significant upward trend as DCP levels rose, hinting at its highest point in the DCP80 group. GST helps attach GSH to electrophilic compounds, aiding their detoxification and removal [52,53]. The marked rise in GST-A under high DCP inclusion may be a compensatory response to potential increased oxidative stress and exposure to foreign substances. While higher DCP diets might introduce more residual gossypol, the lack of analyzed free gossypol data in the finished diets precludes a direct causal link. Instead, this detoxification response may stem from a broader array of dietary stressors, including other plant-derived antinutritional factors or metabolic burdens arising from subtle amino acid imbalances, which must be cleared through the GSH conjugation route [54]. The thioredoxin (Trx) system, comprising *Trx* and thioredoxin reductase (*TrxR*), constitutes a separate antioxidant system that keeps protein thiol groups reduced and helps regulate redox signaling within cells [55]. In our results, both *Trx* and *TrxR* expression levels fell significantly in the higher DCP groups, with the lowest values again found in the DCP80 group. The parallel decline of *Trx* and *TrxR* suggests that the thioredoxin-dependent antioxidant route may also be compromised by higher DCP substitution.

In conclusion, suitable DCP substitution levels (≤40%) maintained relatively stable intestinal antioxidant gene expression, while excessive DCP levels (≥60%) significantly impaired the intestinal antioxidant defense system by downregulating *SOD1*, *Gpx4α*, *GR*, *Gclc*, *Gclm*, *Trx*, and *TrxR*, with concurrent upregulation of GST-A as a compensatory detoxification response. Consistent with these findings, dietary supplementation with low-level cottonseed protein concentrate did not affect intestinal antioxidant capacity in common carp (*Cyprinus carpio*), while higher inclusion levels (75% and 100%) impaired intestinal antioxidant capacity, as reported by Fan et al. (2024) [56]. Similar findings are also found in Asian red-tailed catfish (*Hemibagrus wyckioides*) [57] and juvenile tilapia (*Oreochromis niloticus*) [58]. These results provide preliminary theoretical support for determining appropriate DCP inclusion levels in largemouth bass feeds and highlight the importance of monitoring intestinal antioxidant status when formulating high-plant-protein diets for carnivorous fish species.

### 4.4. Intestinal Barrier Integrity

Given that excessive oxidative stress is known to disrupt epithelial integrity, we then examined whether the compromised antioxidant capacity induced by high DCP levels might lead to physical damage of the intestinal barrier. As a crucial defense against pathogens, the intestinal mechanical barrier comprises epithelial cells and intercellular connections [59]. However, its integrity can be compromised by dietary plant proteins, which have been shown to cause physical damage to the intestinal mucosa in numerous fish species such as juvenile common carp [60], Channa argus [15]. Studies on pike perch (*Sander luciperca*) [61] and largemouth bass [62] have found that when cottonseed protein concentrate replaced a higher proportion of FM, the villus height tended to decrease. In our study, dietary DCP replacement caused alterations in intestinal morphology, with muscular thickness decreasing progressively and being significantly reduced at the highest replacement level, suggesting that excessive DCP inclusion (≥60%) may compromise intestinal structural integrity. Moreover, tight junctions, composed of proteins like occludin, claudins, and ZO-1, form a continuous belt-like structure across the intestinal epithelium, acting as a key barrier [61]. Their structural integrity correlates closely with intestinal villus morphology (linked to villus height and density), while mucin—secreted by goblet cells—cooperates with the epithelial barrier to form intestinal protection [63]. Consistent with the findings on villus height, 80% DCP replacement significantly downregulated the expression of *Mucin-2*, *Mucin-5AC*, *Mucin-13*, *Mucin-17*, *Cldn15*, *Cldn-34* and *Occludin 1*. At the 20% replacement level, only *Cldn15* expression was reduced, with no significant changes observed in the other tested genes. These transcriptional changes, together with the morphological observations, may suggest that DCP could impair intestinal barrier integrity in a dose-dependent manner. Comparable outcomes have been reported in Atlantic salmon (*Salmo salar* L.) [64], largemouth bass [65], and olive flounder (*Paralichthys olivaceus*) [66]. Consistent with these observations, a study by Chen et al. (2025) on grass carp given increasing amounts of phytic acid (0–4.0%) showed that high inclusion of this anti-nutritional factor, notably at 3.2–4.0%, lowered *Mucin* expression [67]. Altogether, the current evidence points to a dose-related disruption of intestinal barrier integrity by DCP at the transcriptional and morphological levels. Still, the exact molecular pathways behind this effect need further investigation, and protein-level or functional confirmations are warranted to validate these observations.

### 4.5. Intestinal Inflammatory Responses

A disrupted intestinal barrier often permits translocation of luminal antigens, thereby triggering local inflammation [66,68,69]. Therefore, we next assessed whether the barrier dysfunction caused by excessive DCP substitution might be accompanied by activation of pro-inflammatory pathways. In fish, the intestinal immune response is centrally mediated by cytokines that regulate inflammation [70]. Key pro-inflammatory cytokines among these are *IL-8*, *IL-1β*, and *IL-12β*, which play critical roles in activating immune cells such as T and B cells and driving the inflammatory process [71]. In this study, 20% DCP supplementation did not induce a significant inflammatory response at the transcriptional level, while replacement levels of 40% and above triggered a substantial and sustained up-regulation of key pro-inflammatory cytokines, suggesting the activation of intestinal inflammatory pathways. Previous studies have shown that plant proteins in the feed caused the fish intestine to exhibit an inflammatory response [72,73,74]. A follow-up study on juvenile channel catfish (*Ictalurus punctatus*) further illustrates that this effect is concentration-specific. After an 8-week feeding trial with enzymatic rice protein replacing 0–7.5% of FM, gene expression analysis demonstrated that a 2.5% inclusion significantly down-regulated intestinal *IL-8* mRNA, while a 7.5% inclusion up-regulated both *IL-1β* and *IL-8* [75].

More importantly, our results revealed that higher DCP replacement levels (≥60%) significantly upregulated the expression of Caspase-1 (*Cas-1*) and Caspase-6 (*Case-6*), key executioner caspases in the inflammatory cell death pathway. *Cas-1* is the central protease of the inflammasome complex, responsible for cleaving *IL-1β* and *IL-18* into their mature, biologically active forms, which can induce pyroptosis [76]. The gradually increasing expression of *Cas-1* with rising DCP levels, reaching its maximum in the DCP80 group, provides transcriptional evidence suggesting that excessive DCP may activate the canonical inflammasome pathway. Meanwhile, Case-6 transcript levels increased in a significant dose-dependent manner, peaking in the DCP80 group. As a cysteine-aspartic protease, *Case-6* is centrally involved in the execution phase of programmed cell death. It also contributes significantly to innate immunity and host defense [77]. In our experiment, higher DCP inclusion led to a simultaneous rise in both *Cas-1* and *Case-6* expression, hinting that excessive DCP addition could trigger both canonical and non-canonical inflammatory cell death pathways within the gut of largemouth bass. Interestingly, Gasdermin E (*GSDM-E*) followed a similar rising pattern as *Cas-1* and *Case-6*, with a significant increase in the DCP80 group versus the control. *GSDM-E* is a pore-forming protein that, after being cleaved by caspases (especially *Cas-1* and *Case-6*), drives secondary necrosis or pyroptosis characterized by cell death marked by swelling and rupture of the plasma membrane [78,79]. The fact that *GSDM-E*, *Cas-1*, and *Case-6* were all sharply higher in the DCP80 group may imply that excessive DCP substitution could activate a coordinated pyroptotic cell death program in the intestine of largemouth bass.

Key intracellular sensors such as the NOD-like receptor family pyrin domain containing 3 (*NLRP3*) inflammasome and nucleotide-binding oligomerization domain-containing protein 1 (*NOD1*) can identify a broad spectrum of pathogen-associated molecular patterns (PAMPs) and damage-associated molecular patterns (DAMPs), for example, ROS, oxidized lipids, lysosomal damage, and bacterial peptidoglycan fragments [80]. Specifically, sensor activation recruits the adaptor *ASC* (apoptosis-associated speck-like protein containing a CARD) to form inflammasome complexes, which then activate *Cas-1* and trigger the maturation and release of *IL-1β* and *IL-18*. In our study, *NLRP3*, *NOD1*, and *ASC* all increased with DCP substitution, with significantly higher expression in the DCP60 and DCP80 groups versus the control. This parallel increase in all three sensors at DCP levels of 60% or higher implies that excessive DCP replacement may simultaneously engage multiple pattern recognition receptor pathways within the intestine of largemouth bass.

To sum up, our results show that replacing FM with high levels of DCP (60% or more) triggers substantial intestinal inflammation in largemouth bass at the transcriptional level, likely through a broad immune reaction. In this context, excessive DCP acts as a stressor, boosting the expression of key pattern recognition receptors (NLRP3 and NOD1). These then recruit ASC, which helps coordinate downstream inflammatory signaling. This sequence ultimately leads to a marked rise in pro-inflammatory cytokines (*IL-8*, *IL-1β*, and *IL-12β*) and the initiation of a coordinated cell death pathway, as seen from increased levels of *Cas-1*, *Case-6*, and *GSDM-E*. Overall, these results are consistent with the notion that too much DCP substitution may damage the intestine by jointly activating the NLRP3 inflammasome, the NOD1-mediated NF-κB pathway, and executioner caspase-dependent pyroptosis. This understanding highlights the importance of carefully optimizing DCP inclusion levels to prevent inflammation-related intestinal damage and maintain proper gut health in largemouth bass aquaculture.

### 4.6. Intestinal Immune Function

Persistent intestinal inflammation often ends up suppressing local immunity, affecting both humoral and cellular defense components. With that in mind, we next examined how the inflammatory response caused by high DCP levels influences gut immune status in largemouth bass. The present study demonstrates that partial replacing FM with DCP modulates intestinal immunity in a dose-dependent way. At the 20% replacement level, most humoral immune parameters (C3, C4, IgM, ACP, and LZM) stayed close to control values, indicating good immunological tolerance. Nevertheless, alkaline phosphatase (AKP) activity dropped even at 20% inclusion, suggesting that AKP is a very sensitive marker of mucosal barrier perturbation and dietary stress from plant-based protein [81]. Once DCP inclusion went above 40%, a clear shift toward immunosuppression became evident, marked by notable declines in several key humoral factors (C3, C4, IgM, ACP, and LZM). The lower levels of complement proteins and IgM in the 60–80% groups suggest widespread impairment of both innate and adaptive mucosal defenses, which may leave the fish more vulnerable to opportunistic pathogens [82]. These observations agree with earlier work in other farmed species, such as rainbow trout (*Oncorhynchus mykiss*) and turbot (*S. maximus*), where excessive inclusion of plant proteins has been linked to weakened immune function and increased disease risk [83,84].

At the gene expression level, these functional findings were backed up by a clear drop in immune-related genes in the high-DCP groups. Hepcidin (*HEPC*), an essential antimicrobial peptide that regulates iron balance and contributes to innate defense, showed a progressive decline with increasing DCP levels, reaching its lowest point at 80% substitution. This reduction probably compromises the antimicrobial capacity of the intestinal mucosa, aligning with earlier findings in largemouth bass fed other plant protein sources [85,86]. Interestingly, *LZM* and immunoglobulins (*Ig-L* and *Ig-M*) showed a biphasic pattern. Moderate DCP inclusion (20–40%) seemed to boost *LZM* expression, while higher amounts (≥60%) significantly suppressed both *LZM* and the immunoglobulin genes. The fact that *LZM* peaked at the 20% replacement level hints that a low dose of DCP may initially activate the innate immune system. However, under the ongoing stress of high-DCP diets, this compensatory response gets overwhelmed. Similar findings have been reported in other studies where high levels of plant proteins altered the expression of these genes [87,88].

In addition, higher substitution levels of DCP (≥60%) led to a significant decrease in several selenoprotein-related genes (*SEPF*, *SEPK*, and *SEPS*) and the tissue factor pathway inhibitor (*Tfpi2*). Selenoproteins are known to support cellular structure and contribute to immune cell movement [89]. Their reduced expression, together with lower Ig-L and Ig-M levels, points to a breakdown in both the structural integrity and immune function of the intestinal barrier [90,91]. Collectively, these findings show that moderate DCP inclusion (20–40%) is generally well accepted, whereas levels of 60% or higher trigger a broad immunosuppressive response. This underscores the need to strike a careful balance between cost-effective protein replacement and preserving gut immune health in largemouth bass farming.

It should be acknowledged that the present trial was conducted solely on juvenile largemouth bass (initial body weight: 3.45 ± 0.05 g) over a relatively short 8-week feeding period. While our multi-layered mechanistic data provide robust insights into the intestinal barrier integrity, inflammatory responses, and oxidative stress status under graded DCP substitution, the findings may not be directly extrapolated to grow-out or broodstock stages. Moreover, the economic feasibility of DCP as an alternative to FM, including cost-benefit trade-offs and the dynamics of long-term fat accumulation, remains to be systematically evaluated. Therefore, future research should extend the experimental duration to cover the entire production cycle and incorporate comprehensive economic analyses under practical commercial farm conditions. Such investigations will be essential to validate the industrial applicability of DCP and to guide its responsible adoption in aquafeed formulations.

## 5. Conclusions

The results demonstrate that substituting 20% of dietary fish meal (FM) with degossypolled cottonseed protein (DCP) was well tolerated by juvenile largemouth bass, with no detectable adverse effects on growth and intestinal health. Excessive substitution (≥60%) triggers oxidative stress, barrier damage, and NLRP3-mediated inflammation, leading to immunosuppression.

## Figures and Tables

**Figure 1:**
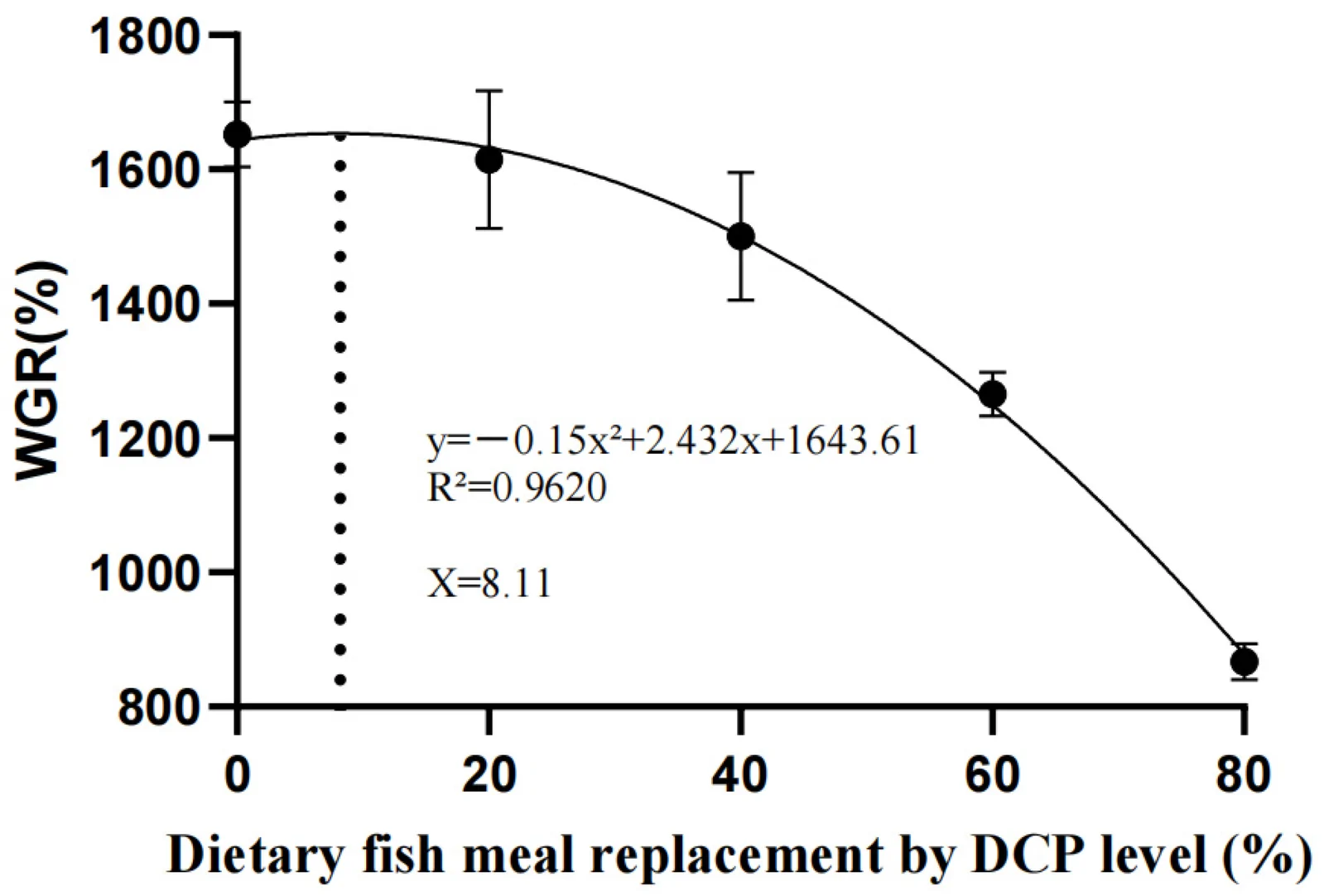
Quadratic regression curve reveals the relationship between replacement level and weight gain rate of largemouth bass.

**Figure 2:**
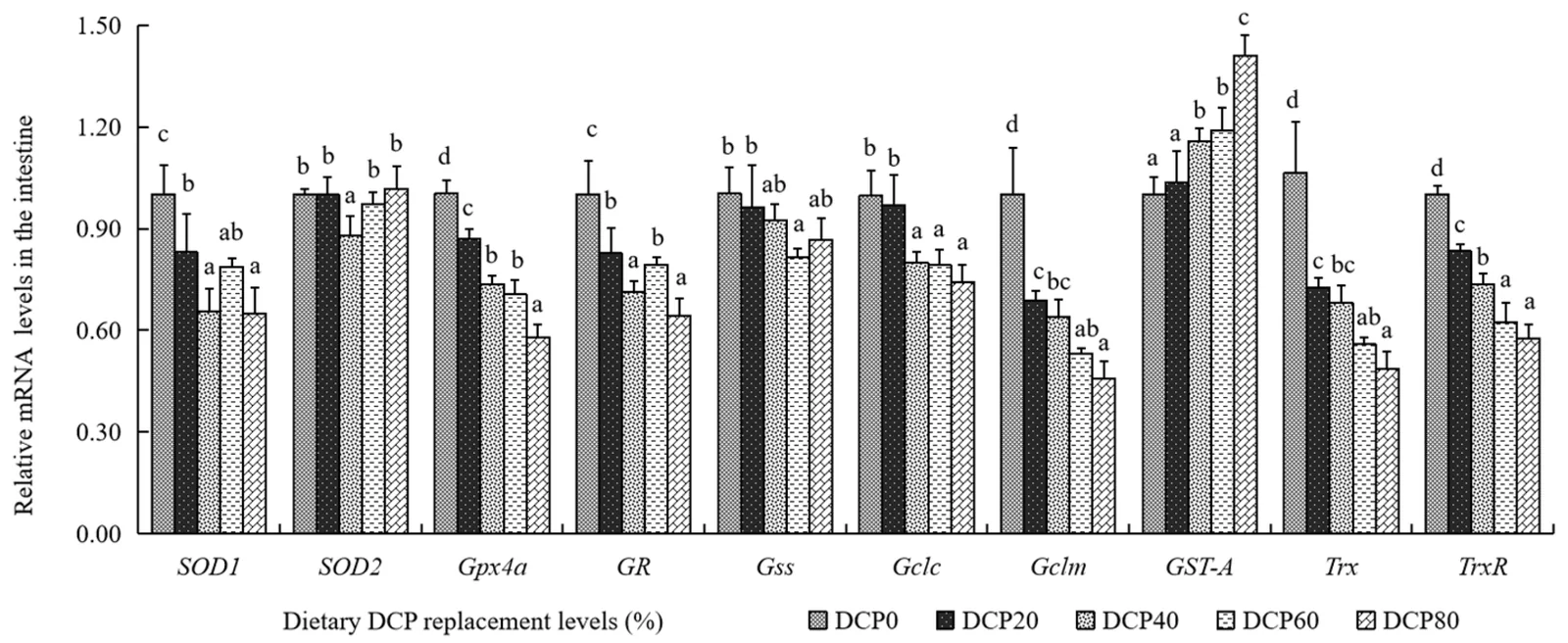
Transcriptional profiles of antioxidant-related genes in the intestine of largemouth bass fed diets with FM substituted by DCP. Data are presented as mean ± SD (standard deviation). Bars bearing different superscripts (a, b, c, d) within the same panel indicate significant differences (*p* < 0.05), whereas bars sharing at least one common superscript letter (e.g., a and ab) are not significantly different (*p* > 0.05).

**Figure 3:**
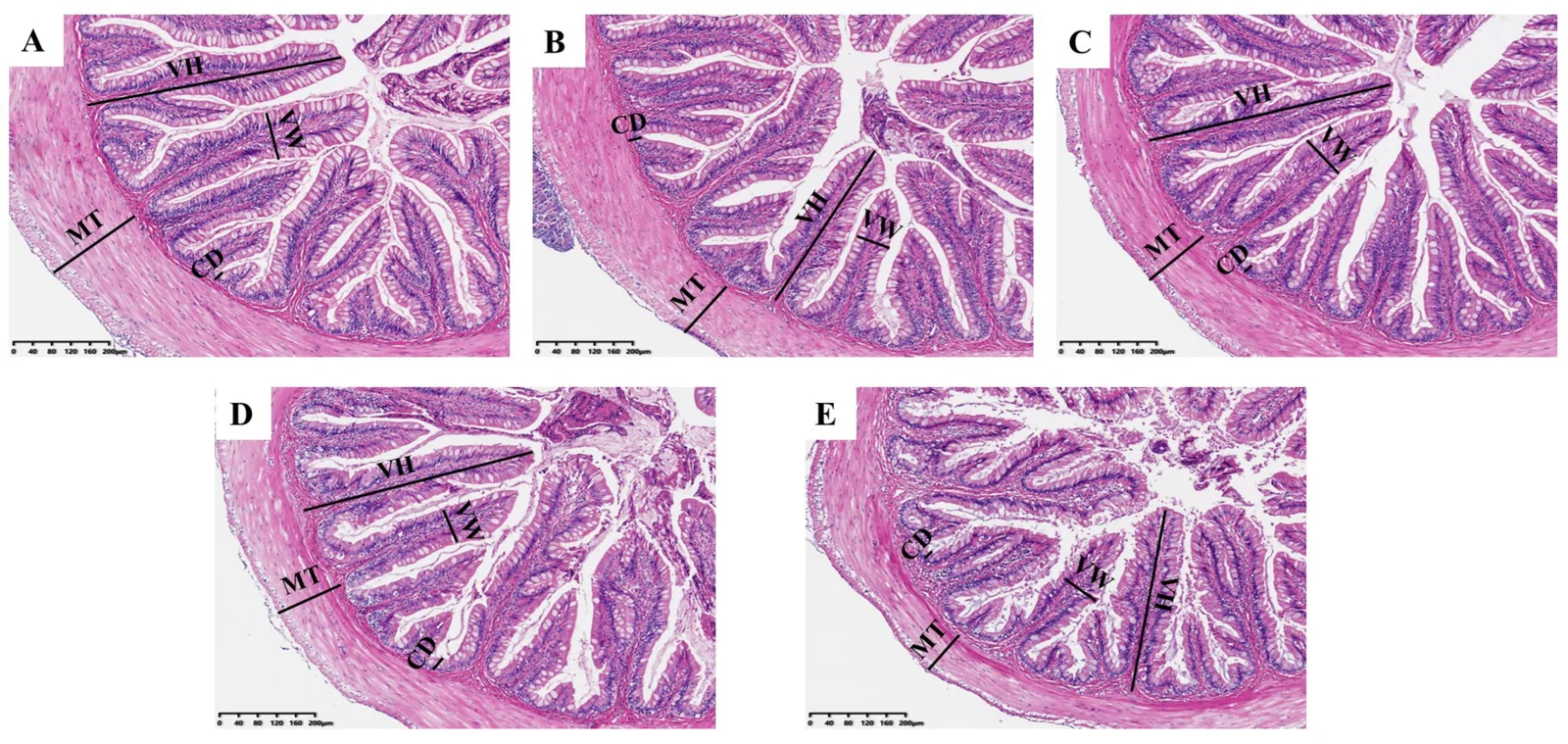
Effects of dietary DCP partial replacing FM on the intestinal morphology of largemouth bass. (**A**) DCP0 (control); (**B**) DCP20; (**C**) DCP40; (**D**) DCP60; (**E**) DCP80. Scale bars = 200 μm. VH, villus height; VM, villi width; MT, muscular thickness; CD, crypt depth.

**Figure 4:**
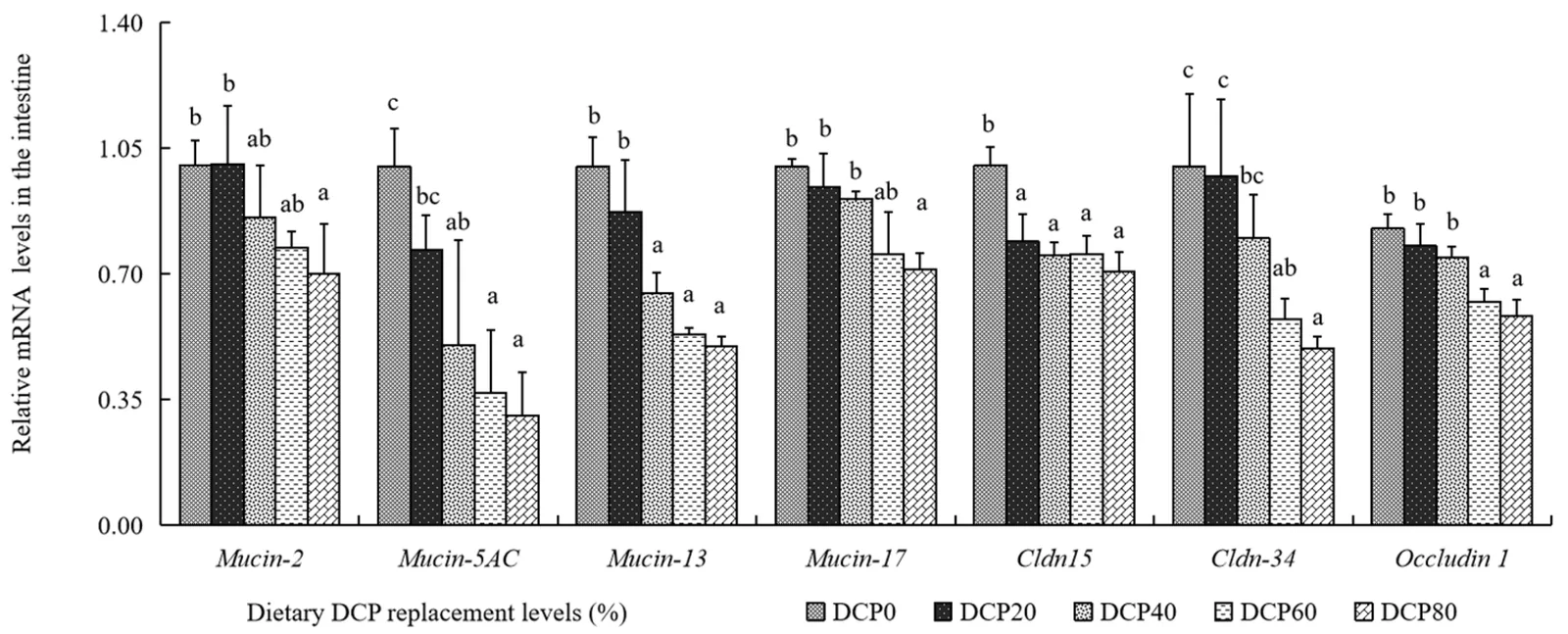
Impact of dietary FM replacement by DCP on the expression levels of barrier-related genes in the intestine of largemouth bass. Data are presented as mean ± SD (standard deviation). Bars bearing different superscripts (a, b, c) within the same panel indicate significant differences (*p* < 0.05), whereas bars sharing at least one common superscript letter (e.g., a and ab) are not significantly different (*p* > 0.05).

**Figure 5:**
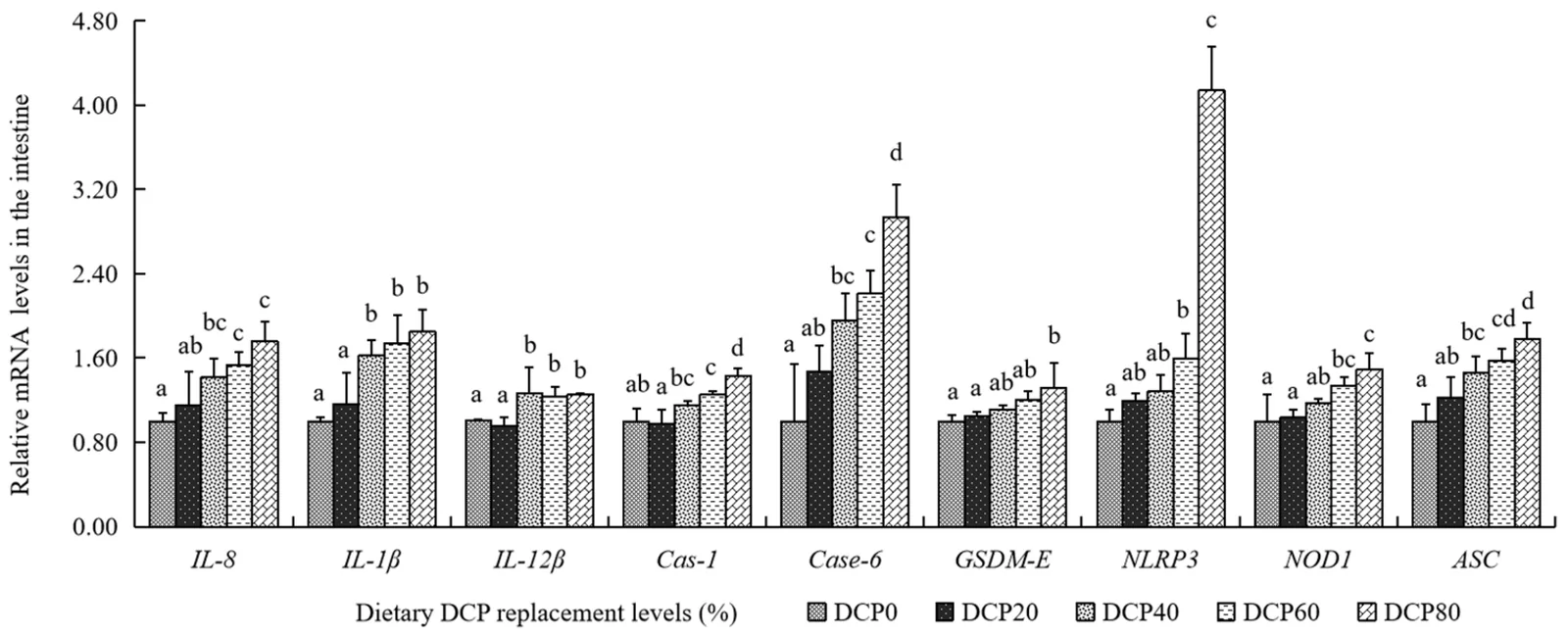
Transcriptional profiles of intestinal inflammation-related genes in largemouth bass fed diets where FM was substituted by DCP. Data are presented as mean ± SD (standard deviation). Bars bearing different superscripts (a, b, c, d) within the same panel indicate significant differences (*p* < 0.05), whereas bars sharing at least one common superscript letter (e.g., a and ab) are not significantly different (*p* > 0.05).

**Figure 6:**
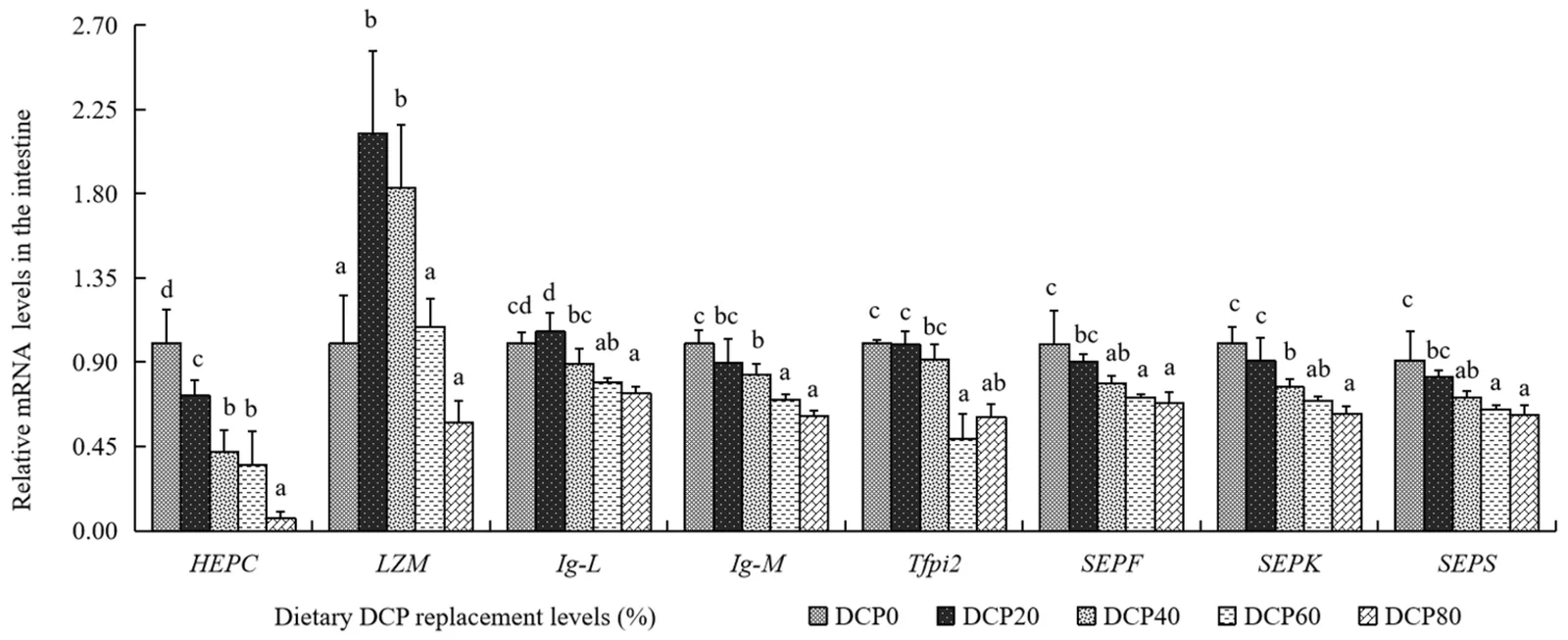
Relative expression levels of intestinal immune-related genes in largemouth bass following replacement of FM with DCP. Data are presented as mean ± SD (standard deviation). Bars bearing different superscripts (a, b, c, d) within the same panel indicate significant differences (*p* < 0.05), whereas bars sharing at least one common superscript letter (e.g., a and ab) are not significantly different (*p* > 0.05).

**Table 1:** Ingredient and proximate composition of diets.

Ingredients	Degossypolled Cottonseed Protein (DCP) Replacement Percentage/%				
	DCP0	DCP20	DCP40	DCP60	DCP80
Fish meal	60.00	48.00	36.00	24.00	12.00
Degossypolled cottonseed protein	0.00	13.20	26.40	39.60	52.80
Soybean meal	10.00	10.00	10.00	10.00	10.00
Casein	3.80	3.80	3.80	3.80	3.80
Dextrin	9.00	9.00	9.00	9.00	9.00
Fish oil	2.20	2.54	2.88	3.23	3.57
Soybean oil	2.20	2.54	2.88	3.23	3.57
Mineral premix ^1^	2.00	2.00	2.00	2.00	2.00
Vitamin premix ^2^	1.00	1.00	1.00	1.00	1.00
Choline chloride	0.40	0.40	0.40	0.40	0.40
Microcrystalline cellulose	9.40	7.52	5.63	3.75	1.86
Total	100.00	100.00	100.00	100.00	100.00
Chemical composition (%)					
Crude protein	48.79	48.13	48.22	48.77	48.58
Crude lipid	11.21	11.34	11.18	11.26	11.37
Moisture	8.19	8.21	8.12	8.51	8.39
Crude Ash	11.85	11.17	10.63	10.12	9.54
Lysine	3.67	3.35	3.03	2.70	2.39
Methionine	1.19	1.07	0.94	0.82	0.69

^1^ Containing the following (mg/kg mineral premix): KI 0.4, CoCl_2_-6H_2_O 52, CuSO_4_-5H_2_O 16, FeSO_4_-7H_2_O 200, ZnSO_4_-H_2_O 280, MnSO_4_-H_2_O 45, MgSO_4_-7H_2_O 1200, Ca (H_2_PO_4_)_2_ 12,000, NaCl 60. ^2^ Containing the following (mg/kg vitamin premix): vitamin A, 20; vitamin D3, 3; vitamin C, 300; vitamin E, 300; thiamin, 20; riboflavin, 10; pyridoxine HCl, 20; vitamin B12, 0.2; vitamin K3, 5; inositol, 1000; pantothenic acid, 30; folic acid, 3; niacin acid, 50; biotin, 1.

**Table 2:** The list of PCR primers used in this study.

Genes	Forward (5′-3′)	Reverse (5′-3′)	Reference
*SOD1*	TGAGCAGGAGGGCGATTC	GCACTGATGCACCCATTTGTA	XM_038708943
*SOD2*	CAGGGATCTACAGGTCTCATT	ACGCTCGCTCACATTCTC	XM_038727054
*Gpx4α*	GCATCATCGTAAATGTAGCCTCT	AGTCCCTGGTTCCTGCCCTC	XM_038718204
*GR*	TGTAAAACGGACAGAGGGCT	CTCGCCGATGTTCAATCCAG	XM_038700350
*Gss*	AGTGAACTGGGAGTGTTTGG	ACGCCTCCGTCTGAGTGT	XM_038723019
*Gclc*	CACCGTGGAGGGCAACAT	AAACCTGGGCAACCTAACC	XM_038737635
*Gclm*	TCTAGTCAACCGGAATCGC	GGAATGGAGCAGTCCAGTGTAT	XM_038715955
*GST-A*	GAGCCCATCAGAACACCC	ACCCAAATAGCACCCAAC	XM_038711179
*Trx*	TTCAGGACCACGATGACTTCAC	TGCAACAGCCTTCTCCAACC	XM_038693856
*TrxR*	GCCTTCTGTAGAGGGAGACA	CTGAACCACCACCAATGAC	XM_038703974
*Mucin-2*	TTGTAATGAGGCCGGTGTCT	CCAGGTGGGGTATAGGGTTG	XM_038706114
*Mucin-5AC*	TGGGCCTTCTAGAGTCTCCT	CTGATTGGTTTAGCGCCCTC	XM_038696526
*Mucin-13*	ATGGGAGCTGTTCGGGCTGT	CATCATCGCTTCTGTCATTTGTG	XM_038736521
*Mucin-17*	AAAGGCGCACAGTTTGGA	TTGGCTTCGACTATGAAGGTAT	XM_038731203
*Cldn15*	CGTGCTCTGATGATTATCTGTC	CTTGGATTGGTCGGTGGC	XM_038718966
*Cldn-34*	GGAACGTCTACTTTGGGA	GGAACTTGATGGTCTGAT	XM_038715311
*Occludin 1*	ATGTGCCAGAACCTGTACCA	GTTTCCCGTAACGCCAGATC	XM_038715419
*IL-1β*	GCGACCGCAGTAAGAAAG	CAGACGGGATAGTCGATGTA	XM_038733429
*IL-8*	TTCTCCTGGCTGCTTTGG	TGGATGGCCCTCCTGTTA	XM_038704093
*IL-12β*	CCGCTGTTATTCAGTCTTACC	GCATCAGGGAGCAGTTCA	XM_038693841
*Cas-1*	ATGAAGCCAGCAGGAAGC	TTGGGATGAGTGCGTTTG	XM_038694367
*Case-6*	ATGCCGTGGACAGTGAGTT	CATACCAGGACCCGTTGAT	XM_038710174
*NLRP3*	GTGCCTTTCAGCATTCACAAC	CATCTTCTTTACCGAGGGCTAG	XM_038736453
*NOD1*	TGTTGGTGGGAGGTATTTG	TGGTAAGACGTGGGTGGT	XM_038712291
*ASC*	AAATAAGGTGGAGGGTAA	AGTCTGCTTCACAGTGGC	XM_038710753
*GSDM-E*	ACATGACGGACGCTACGA	GCTGAAAGGTGCTGGAAA	XM_038711654
*HEPC*	CTCTGCCGTCCCATTCAC	GCATCATCCACGATTCCATT	XM_038710826
*LZM*	CTCTCATTGCTGCCATCA	TGTGTCCACCTCCGTTTG	XM_038713808
*Ig-L*	TATCACTGTGGTGGACTTTTGGT	AGGGGAAGCCCTTGTTGG	NW_024042332
*Ig-M*	AGGTTACCTTCTCCTGCTTG	CTTAGTTCCGTTCTCATAGTTTC	NW_024040041
*Tfpi2*	TGAGCCCTTTAGTTACGGT	GGAATGGAGGCTGAACAC	XM_038724318
*SEPF*	AGGTTCCCTCAAGTCCAAG	CAGCGATGTTCCCGTTAT	XM_038718375
*SEPK*	TGCTTCGTCACGCTTCACT	TCATCCTCCTCCACCCATT	XM_038723206
*SEPS*	GTCTGCTGGTTGTGACTGTGC	TTTCTCCGAGCCGTTTCC	XM_038692445
*β-actin*	TTCACCACCACAGCCGAAAG	TCTGGGCAACGGAACCTCT	XM_038695351

*SOD1*, Cu/Zn-superoxide dismutase; *SOD2*, Mn-superoxide dismutase; *Gpx4α*, glutathione peroxidase 4α; *GR*, glutathione reductase; *Gss*, glutathione synthetase; *Gclc*, glutamate-cysteine ligase, catalytic subunit; *Gclm*, glutamate-cysteine ligase, modifier subunit; *GST-A*, glutathione S-transferase A; *Trx*, thioredoxin; *TrxR*, thioredoxin reductase; *Cldn15*, claudin 15; *Cldn-34*, claudin 34; *IL-1β*, interleukin-1β; *IL-8*, interleukin 8; *IL-12β*, interleukin 12β; *Cas-1*, Caspase-1; *Case-6*, Caspase-6; *NLRP3*, NLR family, CARD domain containing protein 3; *NOD1*, nucleotide-binding oligomerization domain-containing protein 1; *ASC*, apoptosis-associated speck-like protein containing a CARD; *GSDM-E*, gasdermin-E; *HEPC*, hepcidin; *LZM*, lysozyme; *Ig-L*,immunoglobulin light chain; *Ig-M*, immunoglobulin M; *Tfpi2*, tissue factor pathway inhibitor 2; *SEPF*, selenoprotein F; *SEPK*, selenoprotein K; *SEPS*, selenoprotein S.

**Table 3:** Effect of partial replacement of FM with DCP on the growth performance and feed utilization of largemouth bass.

Items	DCP0	DCP20	DCP40	DCP60	DCP80
Initial body weight (IBW)/g	3.49 ± 0.04	3.48 ± 0.07	3.45 ± 0.04	3.47 ± 0.05	3.48 ± 0.05
Final body weight (FBW)/g	61.16 ± 2.35 ^c^	59.68 ± 4.69 ^c^	55.29 ± 3.15 ^c^	47.43 ± 1.74 ^b^	33.67 ± 0.59 ^a^
Weight gain rate (WGR)/%	1652.36 ± 48.88 ^c^	1614.41 ± 102.39 ^c^	1500.71 ± 95.18 ^c^	1265.14 ± 33.14 ^b^	867.03 ± 26.35 ^a^
Specific growth rate (SGR)/(%/d)	4.88 ± 0.08 ^d^	4.73 ± 0.10 ^cd^	4.62 ± 0.10 ^bc^	4.41 ± 0.07 ^b^	3.91 ± 0.05 ^a^
Feed conversion ratio (FCR)	1.00 ± 0.03 ^a^	0.97 ± 0.06 ^a^	1.11 ± 0.05 ^ab^	1.13 ± 0.03 ^ab^	1.28 ± 0.13 ^b^
Viscerosomatic index (VSI)/%	10.24 ± 0.12 ^a^	10.74 ± 0.50 ^ab^	10.62 ± 0.24 ^ab^	11.22 ± 0.12 ^ab^	11.95 ± 1.13 ^b^
Hepatosomatic index (HIS)/%	2.42 ± 0.09	2.40 ± 0.22	2.24 ± 0.06	2.08 ± 0.15	2.14 ± 0.07
Intraperitoneal fat ratio (IPR)/%	3.40 ± 0.04 ^a^	3.18 ± 0.89 ^a^	3.85 ± 0.25 ^ab^	4.67 ± 0.19 ^bc^	5.21 ± 0.37 ^c^
Condition factor (CF)(g/cm^3^)	2.50 ± 0.11	2.43 ± 0.04	2.32 ± 0.06	2.30 ± 0.05	2.45 ± 0.10
Body composition (%)					
Moisture	68.57 ± 0.50 ^a^	69.11 ± 0.28 ^ab^	68.64 ± 0.38 ^ab^	69.14 ± 0.78 ^ab^	69.59 ± 0.36 ^b^
Crude protein	18.01 ± 0.52	18.56 ± 0.23	18.34 ± 0.22	17.92 ± 0.49	17.81 ± 0.29
Crude lipid	9.38 ± 0.52 ^a^	9.94 ± 0.21 ^ab^	9.51 ± 0.42 ^ab^	10.03 ± 0.27 ^ab^	10.52 ± 0.38 ^b^
Ash	3.37 ± 0.05 ^a^	3.38 ± 0.02 ^a^	2.99 ± 0.07 ^b^	2.71 ± 0.02 ^c^	2.28 ± 0.05 ^d^

Note: The data presented are derived from three independent experiments. Within each row, different lowercase letters (e.g., a, b, c, d) denote statistically significant differences at *p* < 0.05, whereas any shared letter (e.g., a and ab) indicates no significant difference (*p* > 0.05).

**Table 4:** Effects of FM replacement by DCP on intestinal digestive enzyme activities of largemouth bass.

Items (U/g Prot)	DCP Replacement Percentage/%				
	DCP0	DCP20	DCP40	DCP60	DCP80
α-amylase (AMS)	0.0850 ± 0.0069 ^b^	0.0952 ± 0.001 ^a^	0.0751 ± 0.0089 ^c^	0.0789 ± 0.0033 ^bc^	0.0398 ± 0.0038 ^d^
Trypsin (TRY)	149.35 ± 7.53 ^a^	290.81 ± 11.28 ^c^	231.64 ± 10.59 ^b^	155.23 ± 7.78 ^a^	138.65 ± 5.09 ^a^
Chymotrypsin (CYT)	8.29 ± 0.93 ^a^	9.88 ± 1.06 ^b^	9.24 ± 0.94 ^ab^	9.04 ± 0.86 ^ab^	8.49 ± 0.69 ^a^

Note: The data presented are derived from three independent experiments. Within each row, different lowercase letters (e.g., a, b, c, d) denote statistically significant differences at *p* < 0.05, whereas any shared letter (e.g., a and ab) indicates no significant difference (*p* > 0.05).

**Table 5:** The effects of FM replacement with DCP on the serum biochemical parameters of largemouth bass.

Ingredients	DCP Replacement Percentage/%				
	DCP0	DCP20	DCP40	DCP60	DCP80
HDL-C (mmol/L)	1.67 ± 0.07 ^ab^	1.66 ± 0.03 ^ab^	1.60 ± 0.01 ^b^	1.67 ± 0.11 ^ab^	1.78 ± 0.06 ^a^
LDL-C (mmol/L)	1.40 ± 0.07 ^a^	1.23 ± 0.03 ^ab^	1.27 ± 0.07 ^ab^	1.13 ± 0.06 ^b^	1.30 ± 0.16 ^ab^
AST (U/L)	53.50 ± 1.49	51.30 ± 2.61	51.77 ± 6.93	49.83 ± 4.96	50.63 ± 3.69
ALT (U/L)	2.03 ± 0.61 ^b^	2.10 ± 0.40 ^b^	2.73 ± 0.15 ^b^	4.43 ± 1.00 ^a^	5.20 ± 0.92 ^a^
ALP (U/L)	143.10 ± 11.53 ^a^	111.60 ± 4.84 ^b^	117.30 ± 6.51 ^b^	104.57 ± 2.81 ^b^	102.97 ± 3.89 ^b^
GLU (mmol/L)	6.99 ± 0.61 ^a^	5.04 ± 0.22 ^b^	3.13 ± 0.14 ^c^	4.59 ± 0.17 ^b^	3.19 ± 0.17 ^c^
TBA (umol/L)	8.33 ± 0.23 ^b^	8.30 ± 0.10 ^b^	8.57 ± 0.12 ^b^	9.23 ± 0.40 ^b^	11.23 ± 1.43 ^a^
ALB (g/L)	8.60 ± 0.10 ^a^	8.47 ± 0.12 ^a^	8.37 ± 0.15 ^a^	8.10 ± 0.26 ^a^	7.57 ± 0.32 ^b^
BUN (mmol/L)	1.27 ± 0.07 ^c^	1.66 ± 0.05 ^ab^	1.58 ± 0.03 ^b^	1.87 ± 0.12 ^a^	1.68 ± 0.14 ^ab^
LDH (U/L)	575.60 ± 44.85 ^b^	662.23 ± 90.71 ^b^	752.73 ± 41.21 ^ab^	746.20 ± 125.64 ^ab^	867.57 ± 57.60 ^a^

Note: The data presented are derived from three independent experiments. Within each row, different lowercase letters (e.g., a, b, c) denote statistically significant differences at *p* < 0.05, whereas any shared letter (e.g., a and ab) indicates no significant difference (*p* > 0.05).

**Table 6:** Effects of dietary DCP levels on intestinal morphology of largemouth bass.

Items	DCP Replacement Percentage/%				
	DCP0	DCP20	DCP40	DCP60	DCP80
Villi height (μm)	465.4 ± 31.3	412.4 ± 28.7	462.1 ± 33.4	471.2 ± 26.8	443.0 ± 14.5
Villi width (μm)	104.6 ± 6.0	102.6 ± 4.7	96.9 ± 4.5	99.2 ± 14.8	81.6 ± 7.6
Muscular thickness (μm)	209.2 ± 5.9 ^a^	154.5 ± 8.6 ^b^	138.3 ± 4.2 ^bc^	134.7 ± 2.8 ^c^	86.4 ± 4.5 ^d^
Crypt depth (μm)	24.5 ± 1.1 ^ab^	19.9 ± 0.3 ^b^	28.7 ± 2.5 ^a^	29.8 ± 2.8 ^a^	22.6 ± 1.3 ^b^
Goblet-cell count (cells/villus)	43.67 ± 2.08 ^a^	40.67 ± 3.21 ^a^	21.33 ± 2.52 ^b^	20.33 ± 2.52 ^b^	18.00 ± 3.60 ^b^

Note: The data presented are derived from three independent experiments. Within each row, different lowercase letters (e.g., a, b, c, d) denote statistically significant differences at *p* < 0.05, whereas any shared letter (e.g., a and ab) indicates no significant difference (*p* > 0.05).

**Table 7:** Impact of dietary FM replacement by DCP on the intestinal humoral immune parameters of largemouth bass.

Ingredients	DCP Replacement Percentage/%				
	DCP0	DCP20	DCP40	DCP60	DCP80
C3 (μg/mg prot)	289.57 ± 23.49 ^a^	297.89 ± 5.79 ^a^	295.25 ± 45.89 ^a^	250.16 ± 22.62 ^b^	233.36 ± 37.51 ^b^
C4 (μg/mg prot)	60.67 ± 3.83 ^b^	65.14 ± 4.50 ^b^	55.21 ± 0.81 ^a^	51.69 ± 1.05 ^a^	52.18 ± 1.78 ^a^
Ig-M (μg/mg prot)	143.5 ± 5.41 ^a^	139.67 ± 6.80 ^a^	140.24 ± 2.62 ^a^	121.43 ± 1.90 ^b^	64.59 ± 9.30 ^c^
AKP (U/g prot)	3.34 ± 0.08 ^a^	2.00 ± 0.02 ^b^	1.89 ± 0.04 ^c^	1.58 ± 0.05 ^d^	1.51 ± 0.07 ^d^
ACP (U/g prot)	22.78 ± 1.52 ^a^	21.56 ± 0.41 ^a^	19.51 ± 0.68 ^ab^	15.17 ± 0.31 ^b^	16.03 ± 0.83 ^b^
LZM (U/mg prot)	322.44 ± 27.01 ^a^	321.15 ± 41.90 ^a^	263.46 ± 27.74 ^b^	268.59 ± 29.12 ^ab^	232.69 ± 15.38 ^b^

Note: The data presented are derived from three independent experiments. Within each row, different lowercase letters (e.g., a, b, c, d) denote statistically significant differences at *p* < 0.05, whereas any shared letter (e.g., a and ab) indicates no significant difference (*p* > 0.05).

## Data Availability

The original contributions presented in this study are included in the article. Further inquiries can be directed to the corresponding authors.

## Abbreviations

The following abbreviations are used in this manuscript:

ACP	Acid phosphatase
AKP	Alkaline phosphatase
ALB	Albumin
ALP	Alkaline phosphatase
ALT	Alanine aminotransferase
AMS	α-amylase
ANFs	Antinutritional factors
ASC	Apoptosis-associated speck-like protein containing a CARD
AST	Aspartate aminotransferase
BUN	Blood urea nitrogen
C3	Complement 3
C4	Complement 4
CF	Condition factor
CK	Creatine kinase
CR	Creatinine
CYT	Chymotrypsin
DAMPs	Damage-associated molecular patterns
DCP	Degossypolled cottonseed protein
FBW	Final body weight
FCR	Feed conversion ratio
FM	Fish meal
Gclc	Glutamate-cysteine ligase, catalytic subunit
Gclm	Glutamate-cysteine ligase, modifier subunit
GLU	Glucose
Gpx4a	Glutathione peroxidase 4α
GR	Glutathione reductase
GSDM-E	Gasdermin-E
GSH	Glutathione
Gss	Glutathione synthetase
GST-A	Glutathione S-transferase A
HDL-C	High-density lipoprotein cholesterol
HEPC	Hepcidin
HSI	Hepatosomatic index
Ig-L	Immunoglobulin light chain
Ig-M	Immunoglobulin M
IL-12β	Interleukin-12β
IL-1β	Interleukin-1β
IL-8	Interleukin-8
IPR	Intraperitoneal fat ratio
LDH	Lactate dehydrogenase
LDL-C	Low density lipoprotein cholesterol
LZM	Lysozyme
NLRP3	NLR family, CARD domain containing protein 3
NOD1	Nucleotide-binding oligomerization domain-containing protein 1
PAMPs	Pathogen-associated molecular patterns
ROS	Reactive oxygen species
SEPF	Selenoprotein F
SEPK	Selenoprotein K
SEPS	Selenoprotein S
SGR	Specific growth rate
SOD1	Cu/Zn-superoxide dismutase
SOD2	Mn-superoxide dismutase
TBA	Total bile acids
TC	Total cholesterol
Tfpi2	Tissue factor pathway inhibitor 2
TG	Triglyceride
Trx	Thioredoxin
TrxR	Thioredoxin reductase
TRY	Trypsin
VSI	Viscerosomatic index
WGR	Weight gain rate
